# Increased Mast Cell Counts in Benign and Malignant Salivary Gland Tumors

**DOI:** 10.5681/joddd.2014.003

**Published:** 2014-03-05

**Authors:** Zohreh Jaafari-Ashkavandi, Mohammad-Javad Ashraf

**Affiliations:** ^1^Associate Professor, Department of Oral and Maxillofacial Pathology, School of Dentistry, Shiraz University of Medical Sciences, Shiraz, Iran; ^2^Associate Professor, Department of Pathology, School of Medicine, Shiraz University of Medical Sciences, Shiraz, Iran

**Keywords:** Adenoid cystic carcinoma, mast cell, mucoepidermoid carcinoma, pleomorphic adenoma, salivary gland tumor.

## Abstract

***Background and aims.*** Mast cells are one of the characteristic factors in angiogenesis, growth, and metastatic spread of tumors. The distribution and significance of mast cells in many tumors have been demonstrated. However, few studies have evaluated mast cell infiltration in salivary gland tumors. In this study, mast cell counts were evaluated in benign and malig-nant salivary gland tumors.

***Materials and methods.*** This descriptive and cross-sectional study assessed 30 cases of pleomorphic adenoma, 13 cases of adenoid cystic carcinoma, 7 cases of mucoepidermoid carcinoma (diagnosed on the basis of 2005 WHO classifica-tion), with adequate stroma in peritumoral and intratumoral areas, and 10 cases of normal salivary glands. The samples were stained with 5% diluted Giemsa solution and the average stained cell counts were calculated in 10 random microscopic fields in peri- and intra-tumoral areas. Data were analyzed by t-test and Mann-Whitney and Krusskal-Wallis tests.

***Results.*** The average mast cell counts increased in the tumors compared to normal salivary glands. There was no signifi-cant difference between benign and malignant tumors and also between different malignant tumors. Infiltration was signifi-cantly denser in peri-tumoral stroma in both tumoral groups (P = 0.001). Minor salivary glands contained significantly more numerous mast cells.

***Conclusion.*** Although mast cell counts increased in benign and malignant salivary gland tumors, there were no signifi-cant differences between the tumoral groups. Further studies are suggested to determine the type of these cells which might be useful in the assessment of biological nature of the tumor and its future treatment modality.

## Introduction

Mast cells (MCc) are bone marrow-derived inflammatory cells which are characterized by their remarkable cytoplasmic granules.^1 ,2^ These cells are present in almost all human tissues (excluding avascular tissues such as bone and cartilage) and are in association with connective tissue structures.^3^ MCs have a multifunctional and complex role in allergic and cell-mediated immune responses, as well as protective antimicrobial reactions.^1,2^ Accumulation of MCs in peritumoral stroma was first described by Westphalia in 1891.^4^ Increasing evidence suggests that MCs have a crucial function in tumorogenesis and tumor growth. Various mediators in the MCs’ granules exert promoting or inhibitory effects on malignancies directly or indirectly.^5^ In vivo and in vitro research studies have shown that MCs release some mediators such as tryptase, chymase, vascular endothelial growth factor (VEGF), fibroblast growth factor-2 (FGF-2) and transforming growth factor-β (TGF-β) which stimulate fibroblast proliferation, fibrosis and angiogenesis.^6-8^ MCs are divided into two major types: tryptase-positive cells (MCT) and also tryptase-chymase-positive cells (MCTC). Tryptase is a strong growth factor for epithelial cells. MCT may upregulate neovascularization in neoplasms. Moreover, MCTC has a role in angiogenesis and fibrosis.^6,7^ Moreover, the above proteases have the ability to degenerate extracellular matrix (ECM) and result in tumor expansion.^9,10^ Some authors have suggested that MC could be a target of cancer therapy. C-kit plays an important role in the growth and function of MCs in mice. Some tyrosine kinase inhibitors used as anti-cancer drugs are potent inhibitors of c-kit. It has been suggested that these drugs act through ablation of MCs.^11^Due to the important effects of MCs on tumoral cells and stroma, MC count (MCC) and density have been evaluated in several human neoplasms. Some previous studies have demonstrated a positive correlation between high MCC and poor prognosis in melanoma, oral squamous carcinoma (SCC) and prostate cancer.^12-14^ Other studies have reported improved survival rate in breast and ovarian cancer with high MCC.^15,16^ However, few studies have been carried out on the presence and distribu-tion of MCs in salivary gland tumors (SGT) and are limited to the pleomorphic adenoma (PA) and Warthin’s tumor.^17,18^ Recently, MCC has been evaluated in a group of minor salivary gland tumors.^19^ As SGTs are relatively common in the head and neck region, in this study the MC distribution was investigated in the most common benign and malignant SGTs, as well as normal tissues from both minor and major SGs.

## Materials and Methods

In this cross-sectional and analytical study, according to the results of previous studies, 30 cases of PA and 20 cases of malignant tumors, including 13 cases of adenoid cystic carcinoma (AdCC) and 7 cases of mucoepidermoid carcinoma (MEC), were enrolled. The tumors were diagnosed on the basis of WHO 2005 classification of SGTs. Ten cases of normal salivary gland (NSG) were obtained from normal glands that were excised along with other pathologic lesions. The samples were retrieved from the archives of Pathology Department of Khallis Hospital, an ENT center affiliated to Shiraz, University of Medical Sciences, taken from May 2005 to April 2009.

Data about the patients’ age and gender and site of the lesion were obtained from their medical documents. After re-evaluation cases with adequate stroma within and around the tumor mass were selected. Samples with severe necrosis and ulceration were excluded. For identification of MCs, 5-mm sections were prepared from formalin-fixed paraffin-embedded samples and stained with 5% diluted Giemsa solution.

MCs were counted in 10 random microscopic fields (at ×400) in the connective tissue at the invasive front of tumors (5 fields) as well as intra-tumoral stroma (5 fields). The average of MCC was separately measured in peri- and intra-tumoral areas.^12,19^In the normal SGs, MCC was evaluated in connective tissue between the epithelial components and the capsular area. Data were analyzed using SPSS 11 by t-test and Mann-Whitney and Krusskal-Wallis tests. P<0.05 was considered significant.

## Results

**Figure 1.**
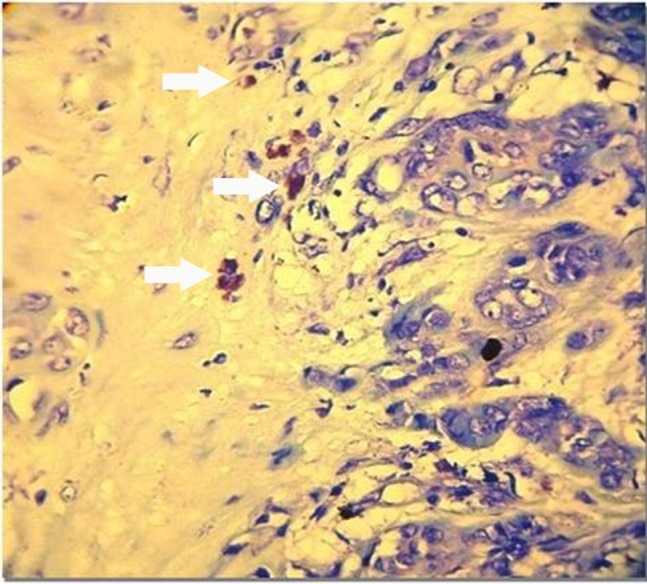
Mast cells with purple granules in pleomor-phic adenoma (Giemsa staining ×200).

**Figure 2.**
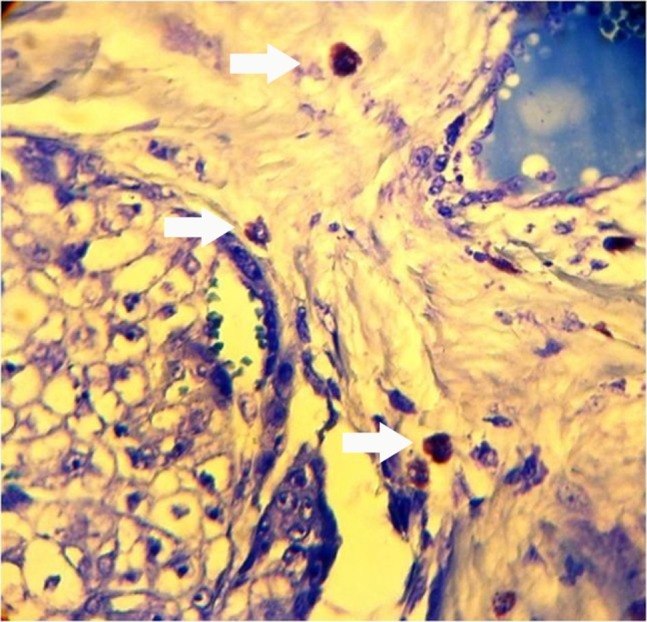
Mast cells with purple granules in mucoepi-dermoid carcinoma (Giemsa staining ×200).

Demographic data about 30 cases of PA, 13 AdCCs, 7 MEGs and 10 NSGs are demonstrated in Table 1. With Giemsa staining, MCs were round, oval or spindle-shaped cells with purple granules (Figures 1,2). All the samples showed mast cell infiltration.

**Table 1:** Baseline data of normal and tumoral samples

Samples	M:F	Age (mean ± SD)	Site Major:Minor
Normal SG (n = 10)	2:8	39.4±15.1	9:1
PA (n = 30)	8:22	36.7±11	28:2
MEC (n = 7)	1:6	52.1±17.9	7:0
AdCC (n = 13)	5:8	49.3±17.3	8:5
Total	16:44	44.4±15.3	52:8
SG: Salivary gland; PA: Pleomorphic adenoma; MEC: Mucoepidermoid carcinoma; AdCC: Adenoid cystic carcinoma.			

In NSGs, they were found sporadically. MCs were located in the capsule as well as adjacent to the ducts and in the fibrous interlobular connective tissue. The mean of MCC was 5.8±9.5 around the salivary glands and 5.1±3.5 in the internal connective tissue.

In the cases of PA, the majority of MCs were found in the capsule and its septa. In the intra-tumoral area, among various types of stroma, fibrous variant comprised a higher number of MCs unlike myxoid, hyalinized and chondroid stroma which appeared to have a lower number of these cells. The mean of MCC in peri- and intra-tumoral stroma was 14.7 ± 7.8 and 7.1 ± 7.2, respectively. Paired t-test showed statistically significant differences between these areas (P = 0.02).

In the cases of AdCC, MCs accumulated around the tumor mass. They were found in the fibrous stroma frequently; however, they were rare in hyalinized stroma. The mean of MCC in peri-tumoral area was 14.1±10.8 and was significantly higher than intra-tumoral part, where the mean of MCC was 4.7 ± 4.4.

In the cases of MEC, the means of MCC were 13.7 ± 7.1 and 5.1 ± 3.5 in peri- and intra-tumoral stroma, respectively, with significant differences (P=0.006).

**Figure 3.**
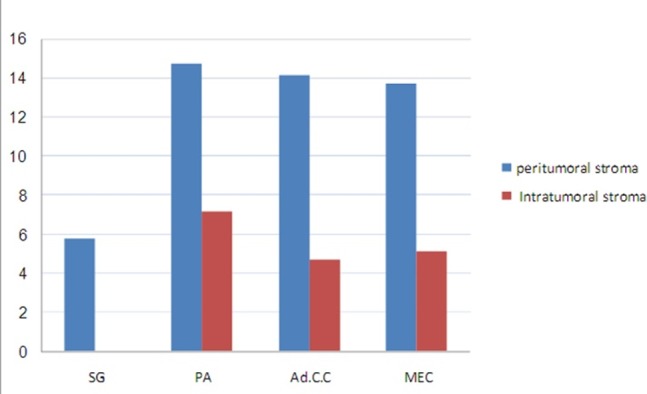
Mast cell counts in peri- and intra-tumoral areas of all groups.

As it is shown in Table 2 and Figure 3, MCC showed an increase in NSGs compared to the tumors. Using Mann-Whitney test, this increase in number was statistically significant when NSGs were compared with PA (P = 0.001), MEC (P = 0.04) and AdCC (P = 0.025). However, this statistical test showed no significant differences between benign and malignant tumors in MCC in peri- and intra-tumoral stroma and also between MEC and AdCC in both areas (P > 0.05). According to the results of Kruskal-Wallis test, there were no significant differences in MCC between the three neoplasms in peri- and intra-tumoral stroma (P > 0.05).

**Table 2:** Mast cell counts in peri- and intra-tumoral stroma of four groups

	Peri-tumoral (mean ± SD)	Intra-tumoral (mean ± SD)
Normal SG (n=10)	5.8±9.3	5.1±3.5
PA (n=30)	14.7±7.8	7.1±7.1
MEC(n=7)	13.7±7.1	5.1±3.5
AdCC (n=13)	14.1±10.8	4.7±4.4
SG: Salivary gland, PA: Pleomorphic adenoma, MEC: Mucoepidermoid carcinoma, AdCC: Adenoid cystic carcinoma		

The mean of MCC in major and minor glands, as well as in males and females, is illustrated in Table 3. The minor SGs showed higher MCCs in comparison with major ones. Mann-Whitney test showed that this difference was significant in peri-tumoral stroma (P = 0.04) and also in intra-tumoral area (P = 0.007). But, there was no correlation between MCC and patients’ gender (P = 0.8).

**Table 3:** Mast cell counts in peri- and intra-tumoral stroma regarding to the site and gender

Variable	Peri-tumoral (mean ± SD)	Intra-tumoral (mean ± SD)
Site		
Major SG (n= 52)	11.6±8.5	5.5±5.5
Minor SG (n= 8)	21.8±8.3	10.7±8
Gender		
Male (n=16)	12.8±9.2	4.4±3.4
Female (n=44)	12.6±8.8	6.9±6.9
SG: Salivary gland		

## Discussion

The results of the present study showed that MCs accumulated around the SGTs and in all the tumoral samples peri-tumoral area presented significantly higher MCC than intra-tumoral stroma. This phenomenon has been found in many other neoplasms such as oral and esophageal SCC, breast cancer, melanoma and cutaneous malignancies.^12,13,15,20-22^.However, studies on MC distribution in the SGTs are limited. Katopodi et al^17^ demonstrated that MCC in PA is greater than canalicular adenoma and many other authors have reported that MCs are present in epithelial cell layer of Warthin’s tumor.^19^ Accumulation of MCs has been attributed to migration of MCs from surrounding connective tissue or from blood vessels.^23^ MC accumulation in this area promotes tumor progression via different pathways, including an increase in angiogenesis, degrading extracellular matrix and basement membrane proteins that facilitate tumor invasion and metastasis.^12,21,22^ However, a study by Jandinski et al^24^ on oral cancers established a slightly lower MCC in high-grade SCC rather than the low-grade one. They justified this reduction as a competition between the immune system and tumor environment. In addition, another study reported a decrease in MCs in OSCC and leukoplakia in comparison with normal oral mucosa due to failure in migration.^25^

In the present study a significant increase was found in MCC in SGTs versus normal tissues, but PAs did not show any significant differences in comparison with carcinomas; even a slight decrease was found in the malignancies. Vidal et al^19^ recently demonstrated higher mast cell counts in benign and malignant minor SGTs. They detected MCs by tryptase antibody using immunohistochemistry (IHC). Their findings also showed no significant differences between benign and malignant tumors. However, Katapodi et al^17^ demonstrated that tumors with modified stroma contained a higher count of MCs. Absence of significant differences in MCC between carcinomas and benign tumors in samples in the present study may be due to different types of stroma in PAs that resulted in increased mean of MCC. Also some authors have reported that intra-tumoral MCs were associated with a favorable prognosis of prostate cancer, whereas peri-tumoral MCs were correlated with poor prognosis.^26,27^ Furthermore, it is possible that the type and function of MCs are different in our benign and malignant SGTs and the special type of MCs in two groups need to be evaluated in further research studies. A study of malignant tumors of breasts showed that MCTs accumulated at the invasive front and chymase-positive cells did not increase.^21^ But Rojas et al,^28^ in comparison of different types of MCs in OSCC, demonstrated that MCT increased in tumoral stroma and MCTC accumulated in peri-tumoral area.

The MC accumulation was seen in the fibrous capsule and stroma of PAs, as well as collagenous stroma of AdCCs and MECs. This result has also been reported by Vidal et al in PA, MEC, AdCC and polymorphous low-grade adenocarcinoma.^19^ Fukushima et al^8^revealed an increased number of tryptase and chymase-positive MCs in fibrotic area in diffuse large B-cell lymphoma. These findings are in agreement with previous results which have shown that MCs directly stimulate fibroblast activation and fibrosis development.^6^ However, Katopodi et al^17^ reported that MCC cannot be related to the type of the stromal connective tissue of PAs as they found different MCCs in the same type of stroma in various cases. In the series of samples in the present study, one case of Pas with extensive chondroid component revealed a large number of MCs, which needs to be interpreted.

According to our findings, there was no difference between MEC and AdCC in MCC, in both peri- and intra-tumoral areas. This result has also been found in minor SGTs.^19^ However, increased MCC was found in peri-tumoral area of both tumors in comparison with intra-tumoral stroma. Comparison between various subtypes of these tumors was not performed because of limited number of cases in the groups. Further studies focusing on special tumors with greater sample sizes should be designed to determine the correlation of MCC with grade and aggressiveness of tumors. In other malignant tumors, most of the authors have demonstrated that MCCs were more numerous than those in normal tissues, reporting controversial results about correlation of NCC with grading of tumors.

Although the sample size from minor salivary glands were limited, our results similar to previous study on PAs showed that MCC in the minor SGTs were more than the major ones.^17^ Also, the results showed similar findings in normal glandular structures. As mucosal MCs accumulate in the subepithelial layer of oral mucosa, where minor SGs are located, it is possible that the difference found could be attributed to the anatomic variation of these neoplasms (major or minor SGs).

In the present study Giemsa solution was used for detection of MCs. Many other researchers have used different types of histostaining such as toluidine blue, Azure and Alcian blue.^12,17,29^ These solutions stain MC granules via metachromatic property. Some authors have used IHC technique for this purpose, reporting similar results about accumulation of MCs around tumors.^4,8,13-15^ It has been shown that both histostaining and IHC techniques are reliable.^29^

## Conclusion

SGTs showed greater MC counts compared to normal SGs but benign neoplasms were similar to malignant ones. MCC counts in minor normal and neoplastic SGs were more than those in major glands, maybe due to anatomical variations. Further studies are suggested to determine the type of MCs in these neoplasms and its relationship to behavior of the tumor.

##  Acknowledgments

This study was funded by the Office of Vice Chancellor for Research of Shiraz University of Medical Sciences (Grant #87-4378). This article is part of the fulfillment for a thesis by Dr. Aman-Allah Akbari. The Authors thank Dr. Shahram Hamedani from the Dental Research Development Center for assistance in the English revision of the manuscript and Dr. Vossoughi for statistical analysis.
